# Effect of different regions on fermentation profiles, microbial communities, and their metabolomic pathways and properties in Italian ryegrass silage

**DOI:** 10.3389/fmicb.2022.1076499

**Published:** 2023-01-16

**Authors:** Zhihui Fu, Lin Sun, ZhiJun Wang, Yichao Liu, Junfeng Hao, Cuiping Gao, Gentu Ge

**Affiliations:** ^1^College of Grassland, Resources and Environment, Key Laboratory of Forage Cultivation, Processing and High Efficient Utilization of Ministry of Agriculture, and Key Laboratory of Grassland Resources, Inner Mongolia Agricultural University, Ministry of Education, Hohhot, China; ^2^Inner Mongolia Academy of Agricultural and Animal Husbandry Sciences, Hohhot, China

**Keywords:** Italian ryegrass, bacterial community, metabolomic profiles, fermentation, different regions

## Abstract

**Introduction:**

Italian ryegrass is less studied in northern China due to high-quality forage grass has not been fully utilized. Full utilization of high-quality forage grass helps to alleviate the shortage of forage grass in winter and spring season and guarantee stable development of livestock production. Consequently, this study was aimed to evaluate the effects of different regions in northern China on the fermentative products, bacterial community compositions, and metabolic pathways and metabolites of Italian ryegrass silage.

**Methods:**

The Italian ryegrass was harvested from three regions (Ordos-WK; Hohhot-AK; Ulanqab-SYK) and ensiled for 60 days. Single molecule real-time (SMRT) sequencing and ultra-high performance liquid chromatography-mass spectrometry (UHPLC–MS/MS) were used to analyze bacterial communities and metabolites, respectively.

**Results:**

After 60 d of fermentation, the SYK group had the lowest pH (4.67), the highest lactic acid contents (95.02 g/kg DM) and largest lactic acid bacteria populations (6.66 log_10_ cfu/g FM) among the treatment groups. In addition, the SYK group had the highest abundance of *Lactiplantibacillus plantarum* (63.98%). In SYK group, isoquinoline alkaloid biosynthesis was the significantly enriched (*p* < 0.05) and high-impact value (0.0225) metabolic pathway. In AK group, tryptophan metabolism the was the significantly enriched (*p* < 0.001) and high-impact value (0.1387) metabolic pathway. In WK group, citrate cycle (TCA cycle) was the significantly enriched (*p* < 0.001) and high-impact value (0.1174) metabolic pathway. Further, *Lactiplantibacillus plantarum* was positively correlated with cinnamic acid, tetranor 12-HETE, D-Mannitol, (2S)-2-amino-4-methylpentanoic acid L-Leucine, guanine, isoleucyl-aspartate and 3,4-Dihydroxyphenyl propanoate, but negatively correlated with isocitrate and D-mannose.

**Discussion:**

In conclusion, this study can improve our understanding of the ensiling microbiology and metabolomics in different regions to further regulate the fermentation products and promote livestock production.

## Introduction

Ensiling has become a common and effective method for long-term conservation of livestock forages. At present, ensiling of forages is widely carried out around the world to continuously provide feeds for ruminants (Wang et al., 2022a). Silage quality largely depends on the characteristics of forage at harvest (Boever et al., 2013), because during the ensiling process, anaerobic microorganisms mainly composed of LAB ferment the available chemical component of the forage and use WSC to produce LA, reduce pH, acidize silage, and inhibit harmful microorganisms to ensure long-term preservation of the silage (Na et al., 2022). The chemical composition of forage is important for silage fermentation (Yin et al., 2022), but the chemical components of forage varies with the environment in which it is grown (Johnson et al., 1999). In addition to the chemical composition, the epiphytic microbiota of the aboveground parts also change with the change of the growing environment (Handley, 2019). However, with the interaction between forage and climate, epiphytic microorganisms gradually become the specific microorganisms of their hosts (Yin et al., 2022). Epiphytic microbial communities are important in ensiling forage during fermentation; composed of a variety of microorganisms, they create a variety of metabolites (Kung et al., 2018).

Italian ryegrass (*Lolium multiflorum* Lam.) is a globally significant fodder crop. It is currently extensively dispersed throughout temperate parts of the world, and cultivated in Europe, America, and Asia (Parvin et al., 2010). Italian ryegrass is one of the most significant and frequent forages for dairy cows in temperate climates (Lv et al., 2021). As the common forage for grazing ruminants, it is characterized by high forage production, nutritional value, digestibility, and good ensiling qualities—it has especially high levels of soluble and degradable nitrogen and carbohydrates (Stergiadis et al., 2015). Making silage from Italian ryegrass can help with bridging the gap between year-round livestock output and the seasonal imbalance of available feed (Wright et al., 2000). However, at present, as a high-quality forage with good ensiling qualities providing high-quality feed for ruminants, Italian ryegrass has been understudied for Italian ryegrass silage in northern China because it is difficult to overwinter in northern areas. Moreover, studies on microbial fermentation and metabolic mechanisms of Italian ryegrass silage have not yet been reported. Therefore, we selected Ordos City, Hohhot City, Ulanqab City, the three representative forage producing areas and milk source gold belt to test the cultivation of high-quality Italian ryegrass to promote the development of high-quality forage industry and dairy industry.

In recent years, SMRT technology has been used to track changing communities of microbial composition and to determine the dominant species in silage, as well as the correlation of dominant species with fermentation products (Bai et al., 2021; Du et al., 2022a). Previous studies have integrated using 16S rRNA sequencing and metabolomics to investigate silage microbiomes and metabolome patterns in order to better understand the biological mechanisms underpinning silage (Guo et al., 2018; Xu et al., 2019). Moreover, many hitherto unknown compounds can be identified by metabolomics (Guo et al., 2018; Xu et al., 2019). Metabolomic research tools help to reveal the biochemical network mechanisms of the fermentation process and can also be used to guide the regulation and prediction of component changes in the fermentation process. During the fermentation process, a significant variety of metabolites are formed, including diverse amino acids, fatty acids, oligosaccharides, vitamins, peptides, and aromatic compounds, and the kinds of these metabolites are researched (Wang et al., 2021). It is important to study the types, quantities, and influencing factors of these metabolites for the exploitation and scientific evaluation of lactic acid bacteria fermentation (Wang et al., 2021). In addition, the use of correlation-based analysis of coupled microbiome-metabolome data sets is becoming increasingly common in research, with the goal of identifying microbial drivers of metabolic variance (Noecker et al., 2019).

To the best of our knowledge, few studies have used multi-omics methods to analyze differences in Italian ryegrass silage fermentation quality, microbial community and metabolome characteristics in different regional settings, and few previous studies have used LC–MS to identify the metabolome of Italian ryegrass silage. We hypothesized that Italian ryegrass grown in different regional environments has different microbial community changes and metabolomics characteristics during fermentation due to differences in chemical composition and microbial composition, resulting in different fermentation quality. Therefore, this study aimed to use SMRT sequencing and metabolomics techniques to study the effects of regions on silage quality from a microbiological and metabolomics perspective. Further, we sought to investigate the microbial community structure and metabolomics characteristics of Italian ryegrass silage fermentation in different regions, so as to better use the regulation of microbial community structure and metabolomics to regulate the fermentation quality to produce high-quality silage. In addition, understanding the relationship between metabolites and fermentation bacteria provides new ideas for the screening and utilization of functional lactic acid bacteria, and provides a theoretical basis for improving fermentation quality.

## Materials and methods

### Experimental design and silage preparation

The planting time, sampling time, and the basic general situation of Italian ryegrass are shown in Table 1. The cultivation and management measures in the three regions are consistent, including irrigation and fertilization. Italian ryegrasses were harvested at booting stages from three plots randomly selected from three regions (WK, AK, SYK) as three replicates, and the collected samples were placed on clean plastic sheets and left to sun-dry. Sun-drying continued until dry matter content was about 35%, whereupon the Italian ryegrass was chopped into 2-cm-long segments with a forage cutter (Mode-8200, Minghong Business, Shandong, China). From each repetition, 300-g samples were collected in sterilized bags (75% alcohol for sterilization) and plated in an ice-boxes, and then immediately sent to the laboratory for determination of microbial community and chemical characteristics (Table 2). The rest of the prepared ryegrass (500 g) was packed into polyethylene plastic bags (size: 300 mm × 400 mm; Embossed Food Saver Bag Co., Ltd., Chengdu, China) and vacuum sealed with a vacuum sealer (DZ-400, Shandong Zhucheng Yizhong Machinery Co., Ltd., Zhucheng, China). There were three replicates for each treatment, and all samples (three treatments × three replicates) were stored at ambient temperature (24–26°C) under sheltered conditions. After 60 days of ensiling, the fermentation quality, microbial community and metabolites of samples from three regions were determined.

**Table 1:** Sampling time and the basic general situation of sample plots.

Items		Regions		
		WK	AK	SYK
Planting time		April 25, 2021	May 12, 2021	June 19, 2021
Sampling time		July 4, 2021	July 7, 2021	August 7, 2021
Elevation above sea level (m)		1242.0	1056.0	1465.0
Geographic position		108°36′E,37°51′N	111°43′E,40°48′N	111°33′E,41°32′N
Monthly mean temperatures (°C)	April	9.4	8.8	6.1
	May	17.1	15.3	12.5
	June	22.2	21.0	18.7
	July	25.2	23.2	20.8
	August	21.4	19.3	17.0
Monthly total precipitation (mm)	April	14.8	30.5	19.1
	May	33.8	7.1	10.8
	June	43.2	55.6	23.8
	July	57.8	122.6	41.2
	August	78.5	111.7	45.8
Monthly mean relative humidity (%)	April	50.6	47.2	51.0
	May	33.3	35.0	37.5
	June	40.4	44.1	41.6
	July	49.1	61.2	62.3
	August	52.3	61.4	61.6
Monthly mean sunshine duration (h)	April	181.7	221.5	148.3
	May	295.6	258.5	256.1
	June	249.4	269.1	281.6
	July	291.0	243.7	135.1
	August	237.5	242.1	263.4

WK, China, Inner Mongolia, Ordos-wushenqi; AK, China, Inner Mongolia, Hohhot; SYK, China, Inner Mongolia, Ulanqab-siziwangqi. Meteorological data sources: www.resdc.cn/.

**Table 2:** Chemical and microbial compositions of fresh materials.

Items	Regions			SEM	*P*-value
	WK	AK	SYK		
DM (%)	33.40b	35.47a	35.04a	1.13	0.101
CP (% of DM)	15.82b	14.65b	23.54a	4.22	<0.001
NDF (% of DM)	58.62a	50.40b	52.53b	4.01	0.028
ADF (% of DM)	33.96a	30.52b	28.21c	2.77	0.017
WSC (% of DM)	7.50b	9.01a	9.23a	0.88	0.028
Lactic acid bacteria (log_10_ cfu/g FM)	4.84	5.06	3.67	0.92	0.210
Coliform bacteria (log_10_ cfu/g FM)	3.68b	2.67b	6.12a	1.67	0.029
Yeast (log_10_ cfu/g FM)	1.26c	3.28a	1.91b	0.93	<0.001
Molds (log_10_ cfu/g FM)	3.34b	4.12a	3.52b	0.38	0.021

FM, fresh matter; DM, dry matter; CP, crude protein; NDF, neutral detergent fiber; ADF, acid detergent fiber; WSC, water-soluble carbohydrate; SEM, standard error of the mean; cfu, colony-forming unit; Means with different letters in the same row (a–c) differ significantly from each other (*p* < 0.05). WK, China, Inner Mongolia, Ordos-wushenqi; AK, China, Inner Mongolia, Hohhot; SYK, China, Inner Mongolia, Ulanqab-siziwangqi.

### Chemical analysis of raw materials and Italian ryegrass silages

Measurement of each key variable (i.e., chemical composition, fermentation characteristics, and microbial counts) of each sample of raw materials and silage was repeated with three replicates. The determination of metabolomics was performed with six replicates. After ensiling, each sample was divided into two equal parts (three replicates × 2 = six replicates). From each sample, 20 g was taken and placed in a sterilized cryopreservation tube. The cryopreservation tube was immediately placed in liquid nitrogen for quick freezing for 15 min, and then stored at −80°C. These samples (three treatments × 6 replicates = 27) were sent to Majorbio Bio-Pharm Technology (Majorbio Bio-Pharm Technology Co. Ltd., Shanghai, China) for metabolomics determination.

Following the method detailed by Du et al. (2021a), the raw materials and Italian ryegrass silages were dried in an oven for 48 h at 65°C until a constant mass was attained, and then ground to pass a 1-mm screen (FM100, Taisite Instrument Co. Ltd., Tianjin, China) for chemical analysis. TN was determined using a Kjeldahl apparatus (Vapodest 50 s, Gerhart, Bonn, Germany) following Patrica (1997), CP content was determined as the TN × 6.25. NDF and ADF were measured according to the method described by Van Soest et al. (1991), using an Ankom A2000i fiber analyzer (A2000i, Ankom Technology, Macedon, NY, United States). The WSC content was analyzed by referring to the anthrone reagent colorimetry Thomas (1977).

In order to analyze the fermentation characteristics of forage, 10 g samples of silage were combined with 90 g of deionized water and kept in a 4°C fridge for 24 h (Du et al., 2021a). The liquid extract was filtered using four layers of gauze and filter paper and used for the following analysis. The pH of the sample was measured with a glass electrode pH meter (pH S-3C, LEICI, Shanghai, China). The organic acid, including LA, AA, PA, and BA content of silage was determined using high performance liquid chromatography (Model: e2695, Waters Corporation, Massachusetts, United States) as described by Cheng et al. (2021). The AN was determined by the phenol-hypochlorite reaction (Broderick and Kang, 1980).

Following Li et al. (2014), 10 g of fresh materials or Italian ryegrass silage was sampled and blended with 90 ml of sterilized water and serially diluted in sterilized distilled water from 10^−1^ to 10^−5^, the numbers of LAB, coliform bacteria, yeast, and molds were then measured. The colonies of LAB were anaerobically cultured on De Man Rogosa Sharpe agar medium at 37°C for 48 h, and the colonies were counted. Coliform bacteria were aerobically incubated at 37°C for 48 h on violet-red bile agar culture media. Yeasts and molds were aerobically incubated at 30°C for 48 h on potato dextrose agar culture media. All culture media were from the same manufacturer (Guangzhou Huankai Microbial Science and Technology Co. Ltd., Guangzhou, China).

### Microbial diversity analysis

The Fast DNA SPIN for Soil kit (MP Biomedicals, Solon, OH, United States) was used to extract genomic DNA from the microbial communities of the fresh and silage ryegrass samples. The concentration and purity of the DNA were evaluated using a Nanodrop 2000 UV–Vis spectrophotometer (Thermo Scientific, Wilmington, DE, United States). The primer pairs 27F and 1492R across the full-length bacterial 16S rRNA gene were amplified using an ABI GeneAmp^®^ 9700 PCR thermocycler (ABI, CA, United States).

The PCR amplification procedure was performed following the methods of Li et al. (2022). The resultant PCR amplicons were extracted from a 2% (w/v) agarose geland further purified using AMPure^®^ PB beads (Pacifc Biosciences, CA, United States). The purified product was mixed in equal molars and a DNA library was constructed using SMRTbell^®^ Express Template Prep Kit 2.0 (Pacifc Biosciences, CA, United States) according to the manufacturer’s instructions. The purified SMRTbell library was sequenced using SMRT sequencing technology on the Pacbio Sequel II System (Pacifc Biosciences, CA, United States). The original 16S rRNA gene sequencing data were spliced using FLASH. UPARSE was used to cluster OTUs with a similarity cutoff of 97%. Each OTU representative sequence was analyzed using the RDP Classifier (Version 2.2) classifier algorithm and database (nt_v20210917), and the confidence threshold was 70%.

Bioinformatic analysis of the silage microbial diversity was carried out using the Majorbio Cloud platform.^1^ Following the methods detailed by Schloss et al. (2009), alpha diversity indices including observed OTUs, Chao1 richness, Shannon index, and Good’s coverage were calculated with Mothur (Version 1.30.1) based on the OTUs information. Following OTU clustering, the Venn diagram package (Version 1.2) in R statistical tools was used to draw a Venn diagram (Du et al., 2021b). The similarity among the microbial communities in different samples was determined by PCoA based on Bray–Curtis dissimilarity using Vegan package (Version 2.5–3). The heat map hierarchical cluster analysis and analysis of similarities (ANOSIM) were shown by R statistical pvclust (Version 3.0.2) (Du et al., 2021b). The distance-based redundancy analysis by Vegan package (Version 2.5–3) was used to examine the relationship between environmental factors and nutrient composition, microbial community structure of the raw materials in three regions (Yang X. et al., 2022).

### Sequencing and analysis of metabolites

As described by Zhang et al. (2019), 50 mg of solid silage ryegrass samples were put in an EP tube and the metabolites were extracted with 400 μl methanol: water (4: 1, v/v) solution. According to the method described by Fu et al. (2022), a mixed quality control sample (QC) was prepared. The processing and testing of QC samples were the same as those of analytical samples. The instrument platform of this LC–MS study used a Thermo Fisher ultra-high performance liquid chromatography tandem Fourier transform mass spectrometry UHPLC-quadrupole-electrostatic field orbital trap high resolution mass spectrometer HF-X system. UHPLC–MS/MS analysis were performed according to the method described by Yang Z. et al. (2022).

### Multivariate statistical analysis of metabolites and identification of differential metabolites

The ropls R package (Version1.6.2) from Bioconductor on Majorbio Cloud Platform (see Footnote 1) was used to perform multivariate statistical analysis, including PCA, PLSDA, and OPLS-DA. The differential metabolites were detected based on OPLS-DA analysis to obtain the first principal component (VIP) of variable importance in the projection. Combined with Student t test, the screening conditions for the main differential metabolites were: *p* < 0.05 and VIP > 1. Using one-way analysis of variance (ANOVA) (*p* < 0.05 for significant difference) and Tukey–Kramer’s test method yielded 471 differential metabolites to create metabolic sets for silage samples from three regions.

Metabolic enrichment and pathway analyses were built up and connected to biochemical pathways using database searches (KEGG).^2^ The KEGG is a knowledge base for systematic analysis of gene function, linking genomic information and functional information. Using the KEGG database, the metabolites in the metabolic concentration can be classified according to the pathways they participate in or the functions they perform. Enrichment analysis is frequently used to analyze a group of metabolites in a function node whether it appears or not. The principle was that the annotation analysis of a single metabolite develops into an annotation analysis of a group of metabolites. KEGG topology analysis was used as the method for relative-betweenness centrality. Scipy.stats (Python packages)^3^ was used to identify statistically significantly enriched pathways using a Fisher’s exact test.

### Statistical analysis

The variance analysis (ANOVA) of fermentation, nutritional characteristics and microbial populations of fresh materials and silage samples were performed using the general linear model program (GLM) to test the differences between samples in SAS software (ver. 9.3; SAS Institute Inc., Cary, NC, United States). One-way analysis of variance (ANOVA) and a Tukey’s honest significant difference (HSD) test were used to examine differences among samples, and the effect was considered significance when *p* < 0.05. Microbiota and metabolome data analysis were performed using the Majorbio I-Sanger Cloud Platform.^4^

## Results

### Chemical composition and microbial population of raw materials

The chemical composition and microbial populations of fresh materials before ensiling are shown in Table 2. The different growth regions had a significant effect (*p* < 0.05) on contents of DM, CP, NDF, ADF, and WSC, as well as coliform bacteria, yeasts and molds numbers. The DM content of fresh materials ranged from 33.4 to 35.47%, and the CP content ranged from 14.65% DM to 23.54% DM. The CP content (23.54% DM) of SYK was significantly higher than the others (*p* < 0.05). The WSC content (9.23% DM) of SYK was the highest and significantly higher than that of WK (*p* < 0.05). There were no significant differences with LAB among the regions, but the LAB numbers of AK were the highest. The counts of undesirable microorganism (e.g., yeast and molds) in WK were the lowest among three groups.

**Figure 1:**
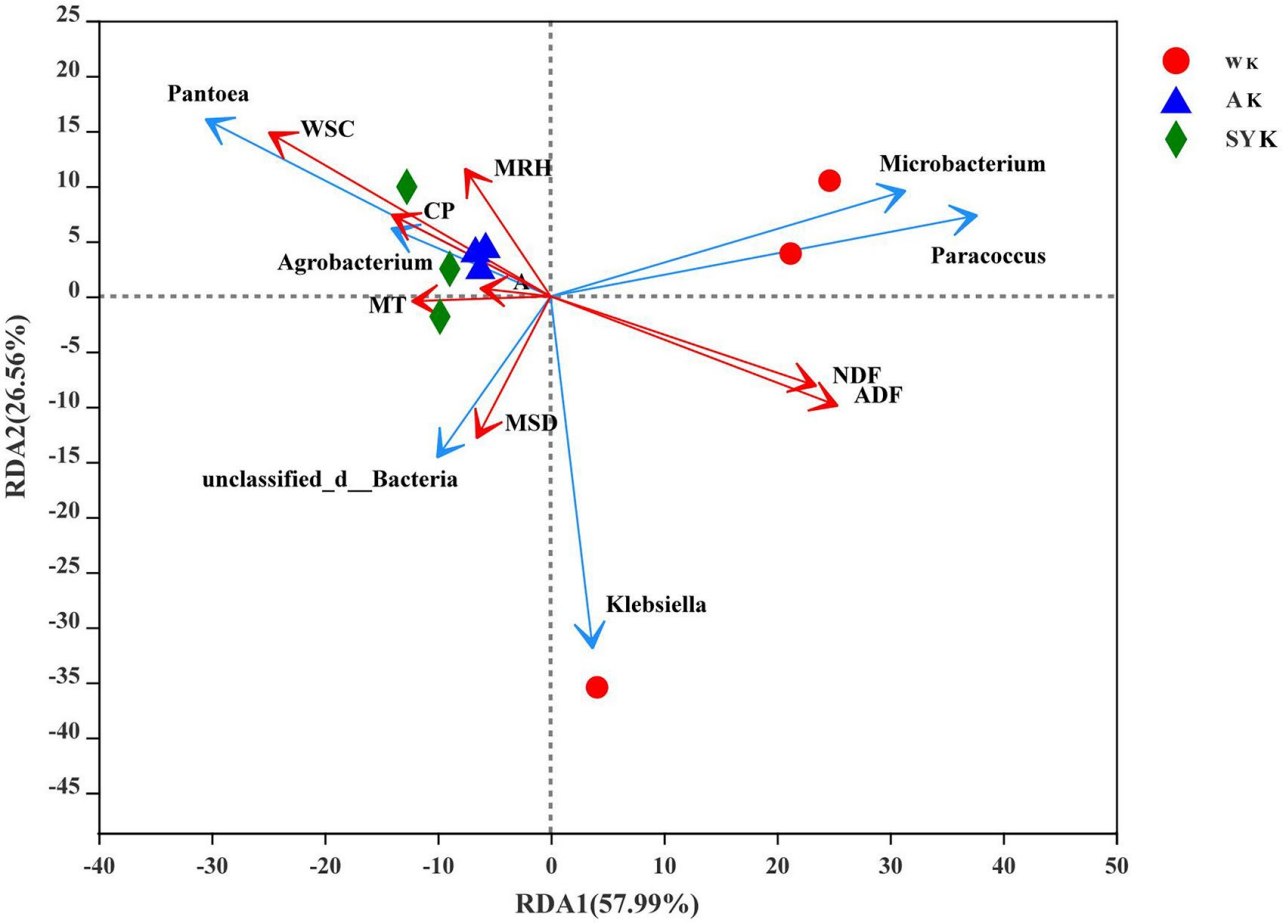
Redundancy analysis the relationship between environmental factors and nutrient composition, microbial community structure of raw materials. The axes are labeled with the percentage of total variance explained (%). Red arrow lengths indicate the variance explained by environmental factors and nutrient composition, and the blue arrow lengths indicate the variance explained with the microbial community structure. Different graphics represent raw material samples of three regions. MT, mean temperature; A, altitude; MRH, mean relative humidity; MSD, mean sunshine duration; CP, crude protein; WSC, water-soluble carbohydrate; NDF, neutral detergent fiber; ADF, acid detergent fiber; WK, China, Inner Mongolia, Ordos-wushenqi; AK, China, Inner Mongolia, Hohhot; SYK, China, Inner Mongolia, Ulanqab-siziwangqi.

To analyze the effect of environmental factors on nutrient composition, microbial community structure of raw materials, redundancy analysis was performed for the microbial communities of fresh materials. Mean relative humidity, WSC, CP, altitude, mean temperature, and mean sunshine duration had a significant effect on the variability of epiphytic bacterial communities. Among them, mean temperature, altitude and mean relative humidity were positively correlated with CP and WSC, and explained up to 84.55% (57.99 and 26.56% on RDA axes 1 and 2, respectively) of total variance of bacterial community (Figure 1).

### Fermentation quality of Italian ryegrass silage in different regions

As shown in Table 3, the regions had a significant effect (*p* < 0.05) on the AN/TN, and the contents of LA, AA, PA, and BA. But we found that there were no significant differences between the numbers of LAB and WSC content after ensiling. The pH of the silages ranged from 4.67 to 5.11, and the SYK achieved lower pH (4.67) as compared to others. Moreover, SYK also had the highest LA content (95.02 g/kg DM). The WK had the highest pH (5.11), while it had the lowest LA content (21.27 g/kg DM) and was significantly lower than the other groups (*p* < 0.05). The SYK had the highest AA (7.77 g/kg DM) and PA (13.35 g/kg DM) content and was significantly higher than the other groups (*p* < 0.05). The LAB population of all three groups increased (4.84 vs. 6.50; 5.06 vs. 5.76; 3.67 vs. 6.66) after 60 days of ensiling, whereas the WSC contents (7.50 vs. 5.25% DM; 9.01 vs. 5.63% DM; 9.23 vs. 5.12% DM) decreased.

**Table 3:** Fermentation quality of ryegrass silage in different regions.

Items	Regions			SEM	*P*-value
	WK	AK	SYK		
pH	5.11a	4.94a	4.67ab	0.23	0.086
AN/TN, %	2.17b	2.35b	3.77a	0.76	<0.001
Lactic acid, g/kg DM	21.27b	54.97ab	95.02a	39.07	0.121
Acetic acid, g/kg DM	0.81c	1.22b	7.77a	3.38	<0.001
Propionic acid, g/kg DM	1.62c	2.21b	13.35a	5.72	<0.001
Butyric acid, g/kg DM	5.04c	9.39a	6.47b	1.95	0.003
Lactic acid bacteria (log_10_ cfu/g FM)	6.50	5.76	6.66	0.54	0.188
WSC (% of DM)	5.25	5.63	5.12	0.49	0.255

FM, fresh matter; DM, dry matter; AN/TN, ammonia nitrogen/total nitrogen; WSC, water-soluble carbohydrate; SEM, standard error of the mean; Means with different letters in the same row (a–c) differ significantly from each other (*P* < 0.05). WK, China, Inner Mongolia, Ordos-wushenqi; AK, China, Inner Mongolia, Hohhot; SYK, China, Inner Mongolia, Ulanqab-siziwangqi.

### Microbial community of raw materials and Italian ryegrass silages

The diversity and richness indices of bacterial communities in raw materials and Italian ryegrass silages are illustrated in Table 4. All of the samples had coverage values above 0.99. The Sobs, Shannon, Ace, and Chao 1 indices in all silage samples were lower than that of all fresh materials. After ensiling, the Sobs, Shannon, Ace, and Chao 1 indices ranged from 31.33 to 47.00, 1.83 to 2.53, 46.01 to 121.75, 44.75 to 85.44, respectively. In addition, the highest values for these indices of bacterial diversity and community were observed in fresh materials of group AK.

**Table 4:** Alpha diversity of the bacterial community in fresh materials and ryegrass silage.

Treatments		Sobs	Shannon	Ace	Chao 1	Coverage
WK	Fresh	92.00	3.00	112.72	109.64	0.9904
	Silage	37.00	2.29	46.01	44.75	0.9962
AK	Fresh	127.67	3.86	143.96	149.41	0.9898
	Silage	47.00	2.53	121.75	85.44	0.9930
SYK	Fresh	97.67	3.48	125.92	120.29	0.9911
	Silage	31.33	1.83	65.93	65.07	0.9953

WK, China, Inner Mongolia, Ordos-wushenqi; AK, China, Inner Mongolia, Hohhot; SYK, China, Inner Mongolia, Ulanqab-siziwangqi.

**Figure 2:**
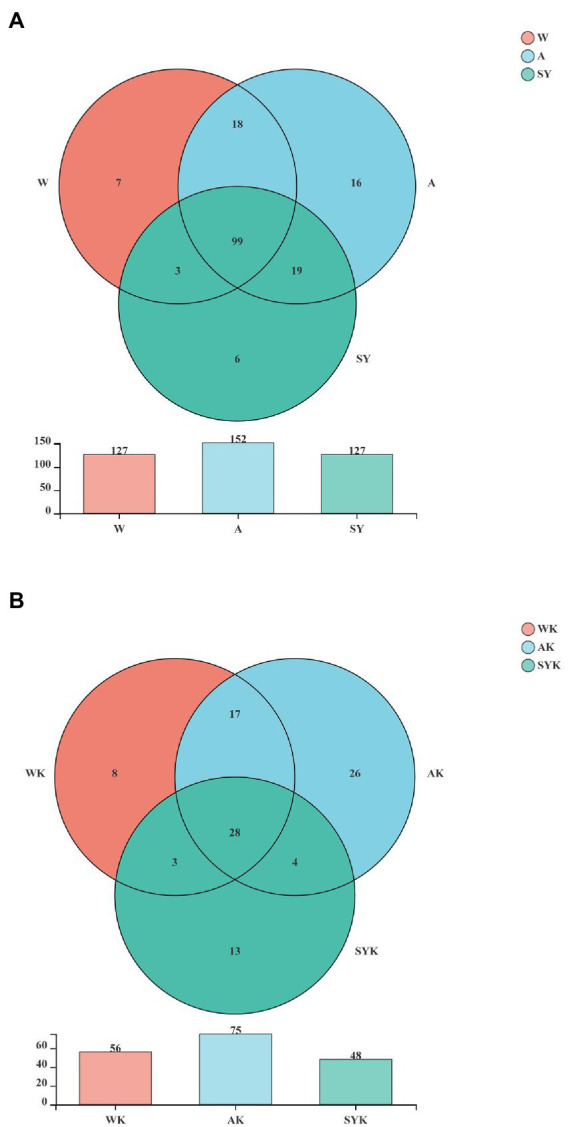
Venn analysis of shared and unique microbial OTUs of raw materials and ryegrass silages. **(A)** Raw materials; **(B)** Ryegrass silages. W, Raw materials of Ordos-wushenqi; A, Raw materials of Hohhot; SY, Raw materials of Ulanqab-siziwangqi; WK, Ryegrass silages of Ordos-wushenqi; AK, Ryegrass silages of Hohhot; SYK, Ryegrass silages of Ulanqab-siziwangqi.

A Venn diagram of the OTUs in raw materials and Italian ryegrass silages is shown in Figure 2. The dominant microbiome of the W, A, and SY (Figure 2A) contained 99 shared OTUs, as well as 7, 16, and 6 unique OTUs, respectively. After ensiling, the WK, AK and SYK silages shared 28 OTUs (Figure 2B), as well as 8, 26, and 13 unique OTUs, respectively. Compared to raw materials, the dominant microbiome of Italian ryegrass silages shared OTUs decreased, and this trend was the same as the Sobs, Shannon, Ace, and Chao 1 indices, indicating that the microbial diversity was decreased after ensiling, while the different regions significantly affected microbial diversity.

**Figure 3:**
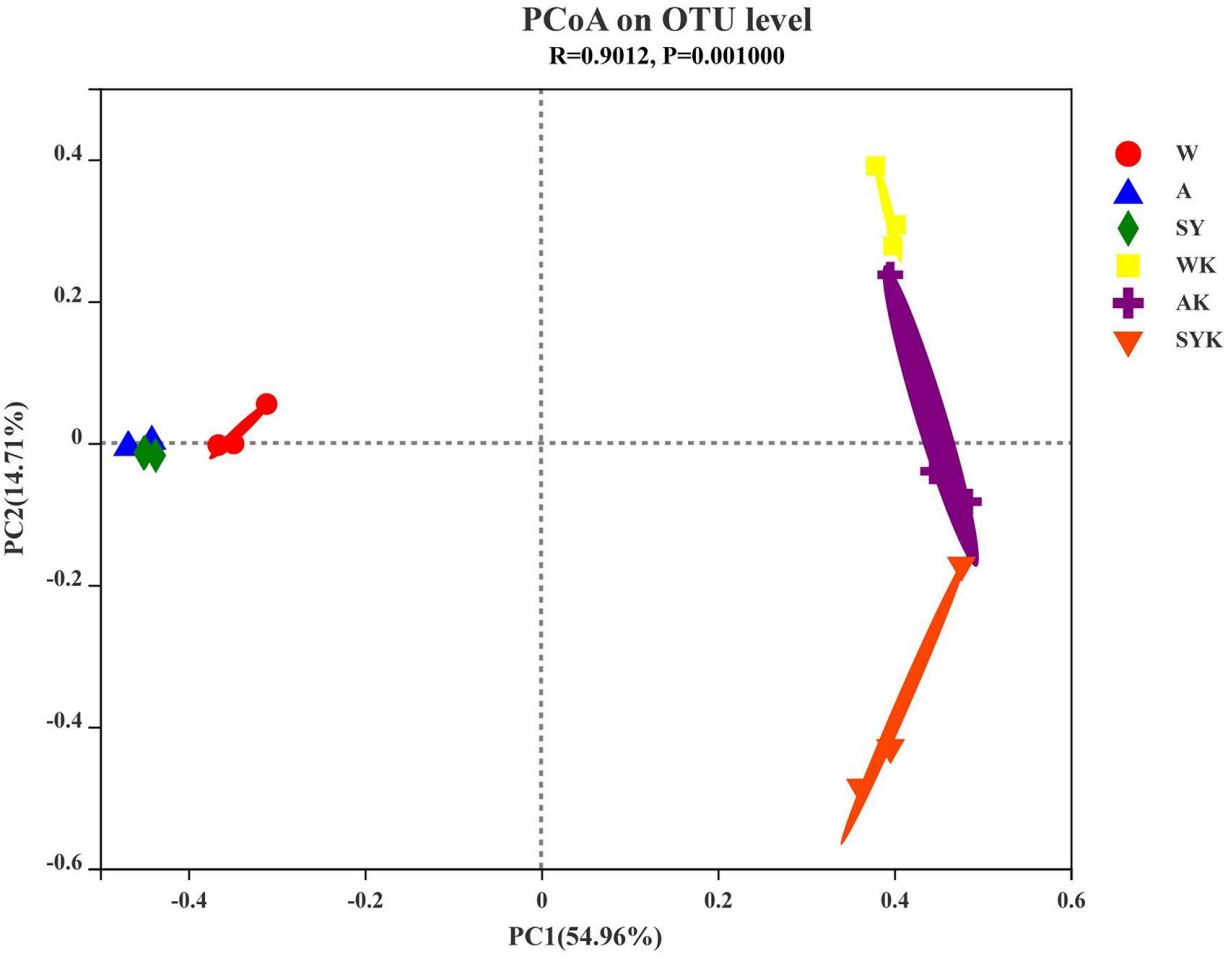
The principal coordinate analysis (PCoA) plot showing variation in bacterial community structure of three regions. W, Raw materials of Ordos-wushenqi; A, Raw materials of Hohhot; SY, Raw materials of Ulanqab-siziwangqi; WK, Ryegrass silages of Ordos-wushenqi; AK, Ryegrass silages of Hohhot; SYK, Ryegrass silages of Ulanqab-siziwangqi.

We used β-diversity analysis utilizing PCoA to confirm differences in the bacterial communities of the fresh materials and Italian ryegrass silage (Figure 3). The PCoA was based on Bray–Curtis distances at the OTU level and an ANOSIM test with 999 permutations between the different groups. As shown in Figure 3, we found that there was no significant separation between bacterial communities of raw materials, but after ensiling we observed a clearly separation among the bacterial communities in different regions, showing that different regions significantly affected the β diversity of microbial communities.

**Figure 4:**
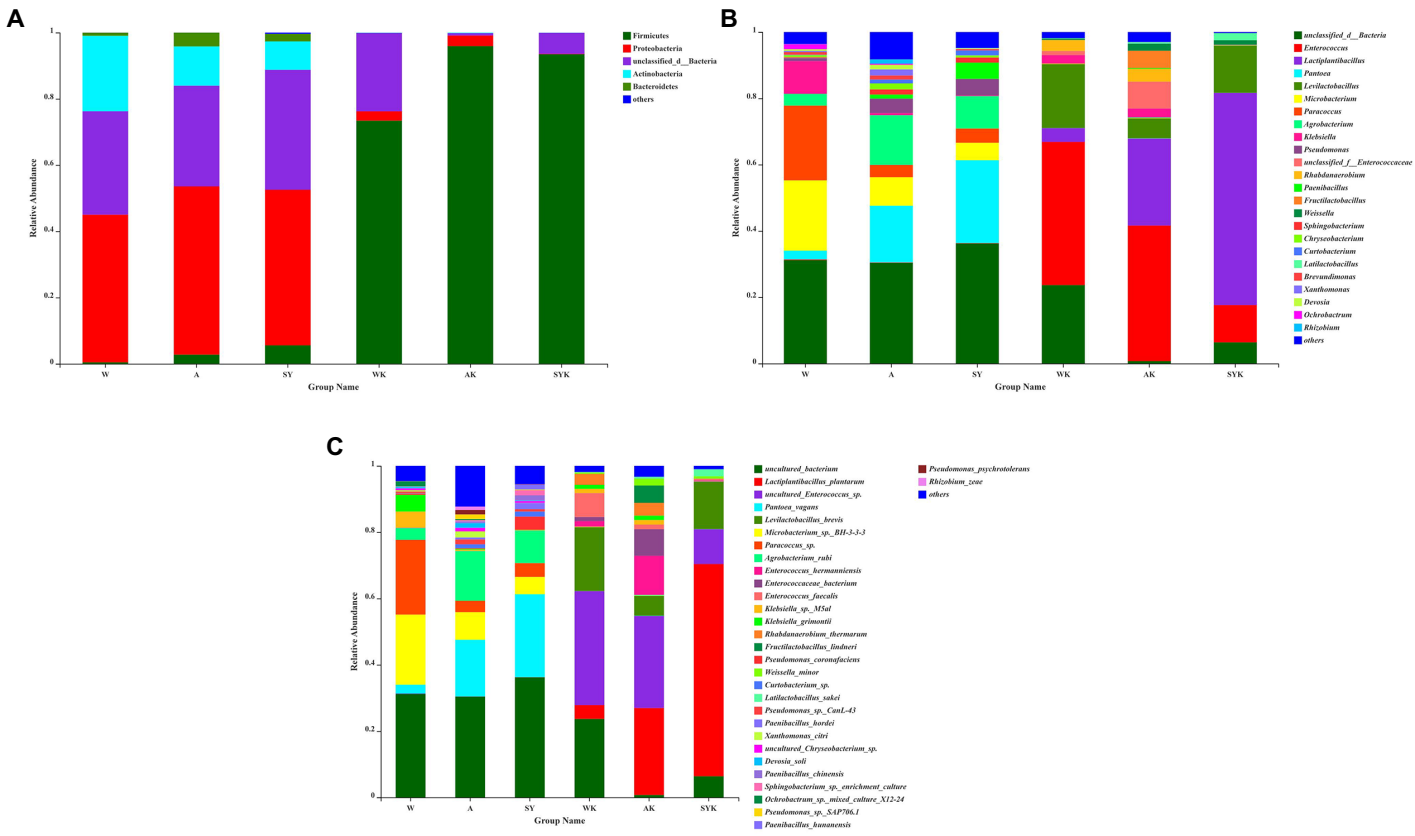
Bacterial community composition on phylum **(A)**, genus **(B)**, and species **(C)** level in fresh ryegrass and ryegrass silage by SMRT. W, Raw materials of Ordos-wushenqi; A, Raw materials of Hohhot; SY, Raw materials of Ulanqab-siziwangqi; WK, Ryegrass silages of Ordos-wushenqi; AK, Ryegrass silages of Hohhot; SYK, Ryegrass silages of Ulanqab-siziwangqi.

Relative bacterial abundances at the phylum (Figure 4A), genus (Figure 4B) and species (Figure 4C) level in raw materials and Italian ryegrass silages are shown in Figure 4. Proteobacteria was the main phylum in the fresh materials, while Firmicutes were the main phylum in Italian ryegrass silages. The relative abundance of Proteobacteria in W, A and SY were 44.57, 50.8, and 46.96%, respectively. After ensiling, the abundance of Proteobacteria decreased while Firmicutes abundance increased. The relative abundance of Firmicutes in WK, AK, and SYK were 73.42, 95.91, and 93.44%, respectively. Unclassified bacteria, *Pantoea*, *Microbacterium*, and *Paracoccus* were the main genera in raw materials. After fermentation, *Enterococcus*, *Lactiplantibacillus*, and *Levilactobacillus* were the most abundant bacteria. The *Enterococcus* relative abundance of WK, AK, and SYK were 43.17, 40.88, and 11.24%, respectively. The *Lactiplantibacillus* relative abundance of WK, AK, and SYK were 4.17, 26.24, and 63.98%, respectively. The *Levilactobacillus* relative abundance of WK, AK, and SYK were 19.28, 6%, and 14.28%, respectively. The highest abundance of *Lactiplantibacillus* occurred in SYK (63.98%) while and the highest abundance of *Enterococcus* occurred in WK and AK (43.17 and 40.88%, respectively). After ensiling, uncultured *Enterococcus* sp. (34.37%) and uncultured bacterium (23.68%) were the predominant species in WK, while the relative abundance of *Lactiplantibacillus plantarum* was only 4.17%. The relative abundance of *Lactiplantibacillus plantarum* and uncultured *Enterococcus* sp. in AK were 26.23 and 27.76%, respectively. The relative abundance of *Lactiplantibacillus plantarum* and uncultured *Enterococcus* sp. in SYK were 63.98 and 10.52%, respectively.

### Correlation analysis of fermentation products and microbial community

**Figure 5:**
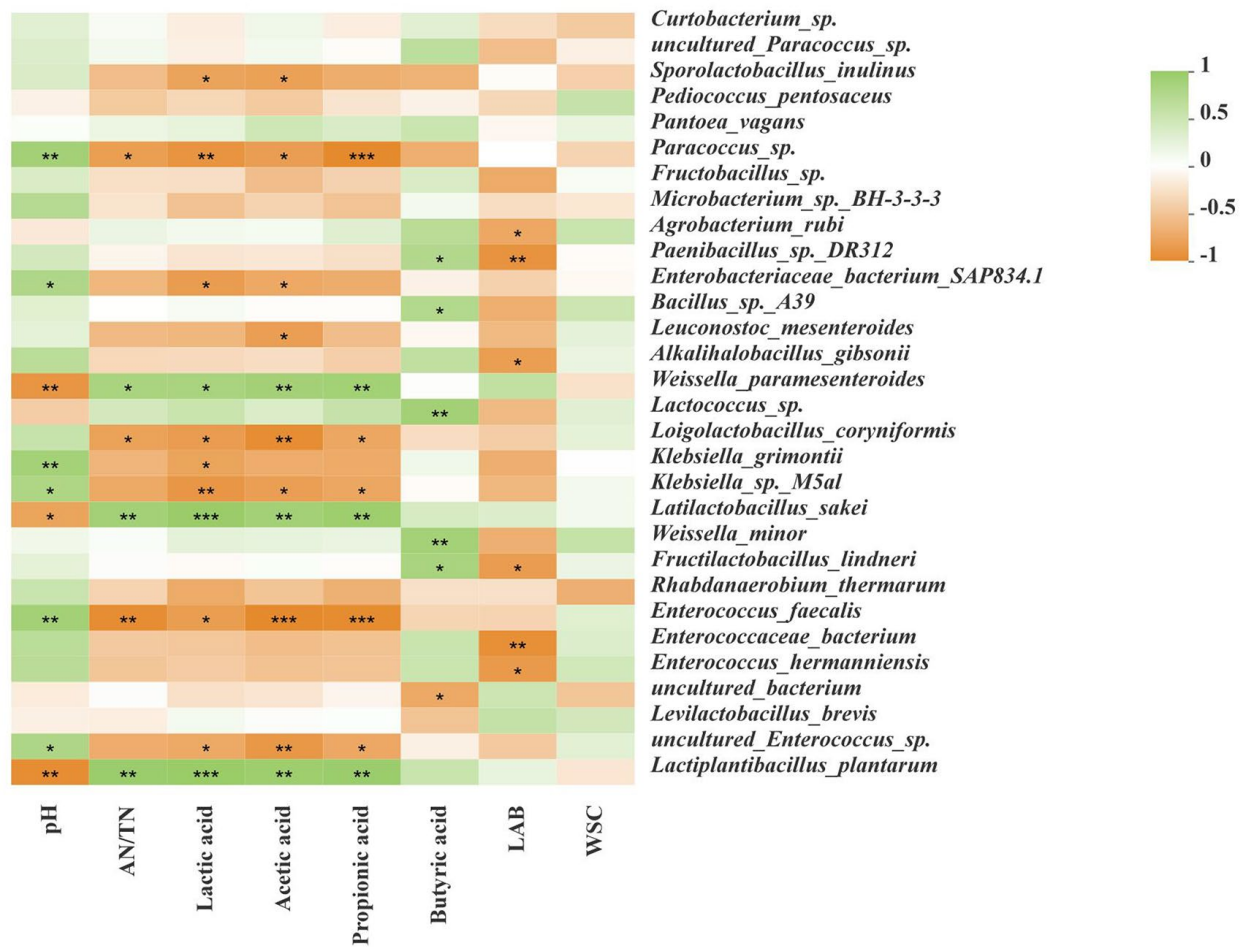
Correlation analyses between bacterial community and terminal fermentation products at species level. Fermentation characteristics are displayed horizontally and the bacterial community information is displayed vertically. The corresponding value of the middle heat map is the Spearman correlation coefficient r, which ranges between −1 and 1; *r* < 0 indicates a negative correlation (orange), *r* > 0 indicates a positive correction (green), and “*,” “**,” and “***” represent *p* < 0.05, *p* < 0.01, and *p* < 0.001, respectively. AN/TN, ammonia nitrogen/total nitrogen; LAB, lactic acid bacteria; WSC, water-soluble carbohydrate.

As shown in Figure 5, the pH was negatively correlated with *Lactiplantibacillus plantarum*, *Latilactobacillus sakei*, and *Weissella paramesenteroides*, but positively correlated with *Enterococcus faecalis* and uncultured *Enterococcus* sp. The AN/TN was positively correlated with *Lactiplantibacillus plantarum*, *Latilactobacillus sakei*, and *Weissella paramesenteroides*, but negatively correlated with *Enterococcus faecalis, Paracoccus* sp., and *Loigolactobacillus coryniformis*. LA, AA and PA were positively correlated with *Lactiplantibacillus plantarum*, *Latilactobacillus sakei*, and *Weissella paramesenteroides*, but negatively correlated with uncultured *Enterococcus* sp., *Enterococcus faecalis*, and *Paracoccus* sp. BA was positively correlated with *Lactococcus* sp., *Weissella minor*, and *Fructilactobacillus lindneri*, but negatively correlated with uncultured bacterium. LAB was negatively correlated with *Agrobacterium rubi*, *Alkalihalobacillus gibsonii*, *Fructilactobacillus lindneri*, and *Enterococcus hermanniensis*.

### KEGG enrichment analysis and topology analysis of metabolite pathway identified in Italian ryegrass silage

**Figure 6:**
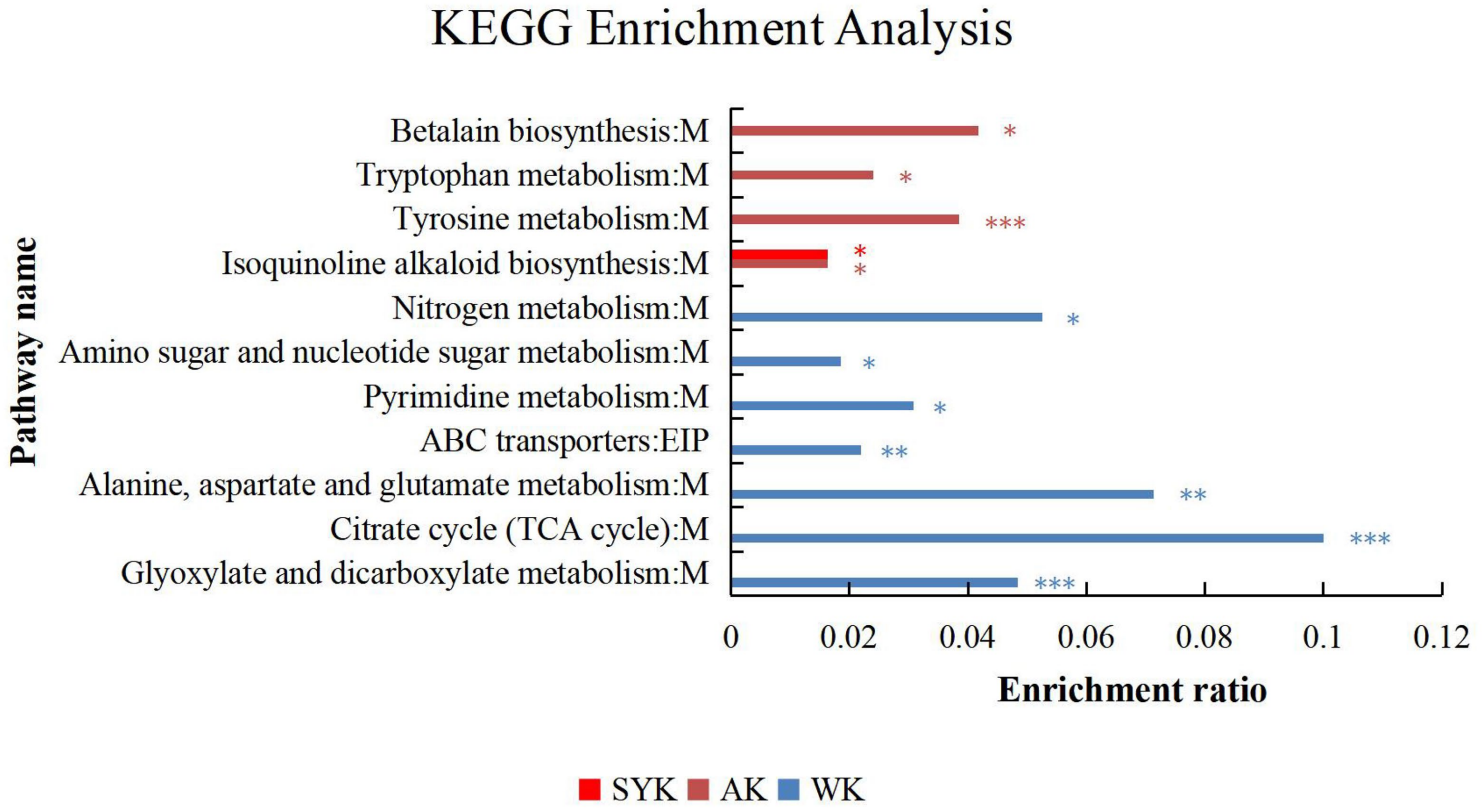
KEGG pathway enrichment analysis of the main differential metabolites of ryegrass silage from three different regions in positive and negative ion modes (WK, Ordos; AK, Hohhot; SYK, Ulanqab). M, and EIP are the class names of metabolic pathways in KEGG annotation. M, metabolism; EIP, environmental information processing. Value of *p*-uncorrected <0.05 and column chart showing value of *p* values for the top 20 pathways; *p < 0.05; **p < 0.01, ***p < 0.001.

KEGG enrichment analysis of differential metabolites after ensiling from three regions in Figure 6. The ordinate in the figure represents the pathway name, and the abscissa represents the enrichment rate, which indicates the ratio of the numbers of enriched metabolites in the pathway to the background number annotated to the pathway. The larger the value, the greater the degree of enrichment. In SYK group, only isoquinoline alkaloid biosynthesis metabolic pathway was significantly enriched (*p* < 0.05), and the enrichment rate was 0.0164. In AK group, there were 4 significantly enriched pathways. They were betalain biosynthesis, tryptophan metabolism, tyrosine metabolism and isoquinoline alkaloid biosynthesis, among which tyrosine metabolism were significantly enriched (*p* < 0.001), and betalain biosynthesis had the highest enrichment rate (0.0417). In WK group, there were 7 significantly enriched pathways. There were nitrogen metabolism, amino sugar and nucleotide sugar metabolism, pyrimidine metabolism, ABC transporters, alanine, aspartate and glutamate metabolism, citrate cycle (TCA cycle) and glyoxylate and dicarboxylate metabolism, among which citrate cycle (TCA cycle) were significantly enriched (*p* < 0.001), and it had the highest enrichment rate (0.1). According to the classification of KEGG pathway database, except for ABC transporters, which are related to environmental information processing, the remaining metabolites belong to the metabolic class.

**Figure 7:**
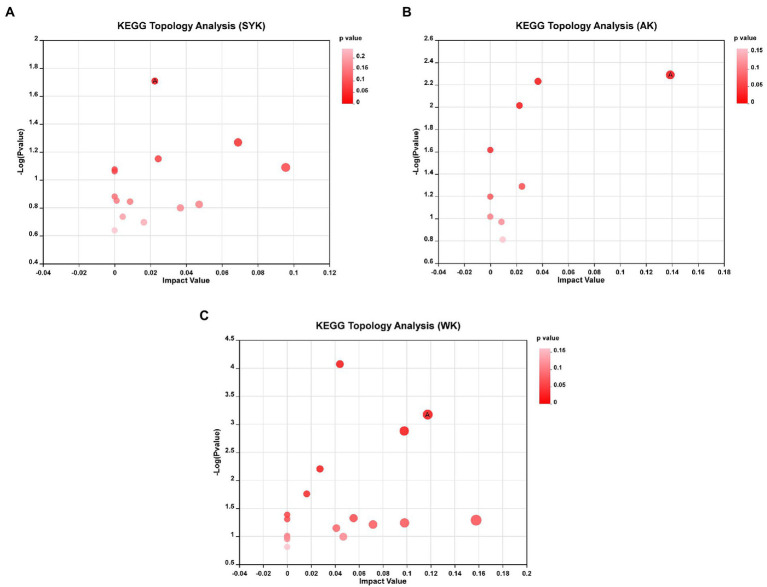
**(A)** SYK, Ryegrass silages of Ulanqab-siziwangqi, **(B)** AK, Ryegrass silages of Hohhot and **(C)** WK, Ryegrass silages of Ordos-wushenqi; Bubbles labeled with the letter A in the figure represent significantly enriched (*p* < 0.05) and high-impact value metabolic pathways.

Topology analysis of differential metabolites after ensiling from three regions as shown in Figures 7A–C, and KEGG topology statistics table was shown in Supplementary Table 1. Each bubble in the figure represents a KEGG pathway; the horizontal axis represents the relative importance of the metabolites in the pathway, the size of impact value; the vertical axis represents the enrichment significance of metabolites involved in the pathway-log10 (*p*-value); the size of the bubble represents the impact value; the larger the bubble, the greater the importance of the pathway. Bubbles labeled with the letter A in the figure represent significantly enriched (*p* < 0.05) and high-impact value metabolic pathways. In SYK group (Figure 7A), the significantly enriched and high-impact value (0.0225) metabolic pathway was isoquinoline alkaloid biosynthesis. In AK group (Figure 7B), the significantly enriched and high-impact value (0.1387) metabolic pathway was tryptophan metabolism. In WK group (Figure 7C), the significantly enriched and high-impact value (0.1174) metabolic pathway was citrate cycle (TCA cycle). This was consistent with the results of KEGG enrichment analysis.

### Correlation analysis of high abundance bacteria and fermentation metabolites

**Figure 8:**
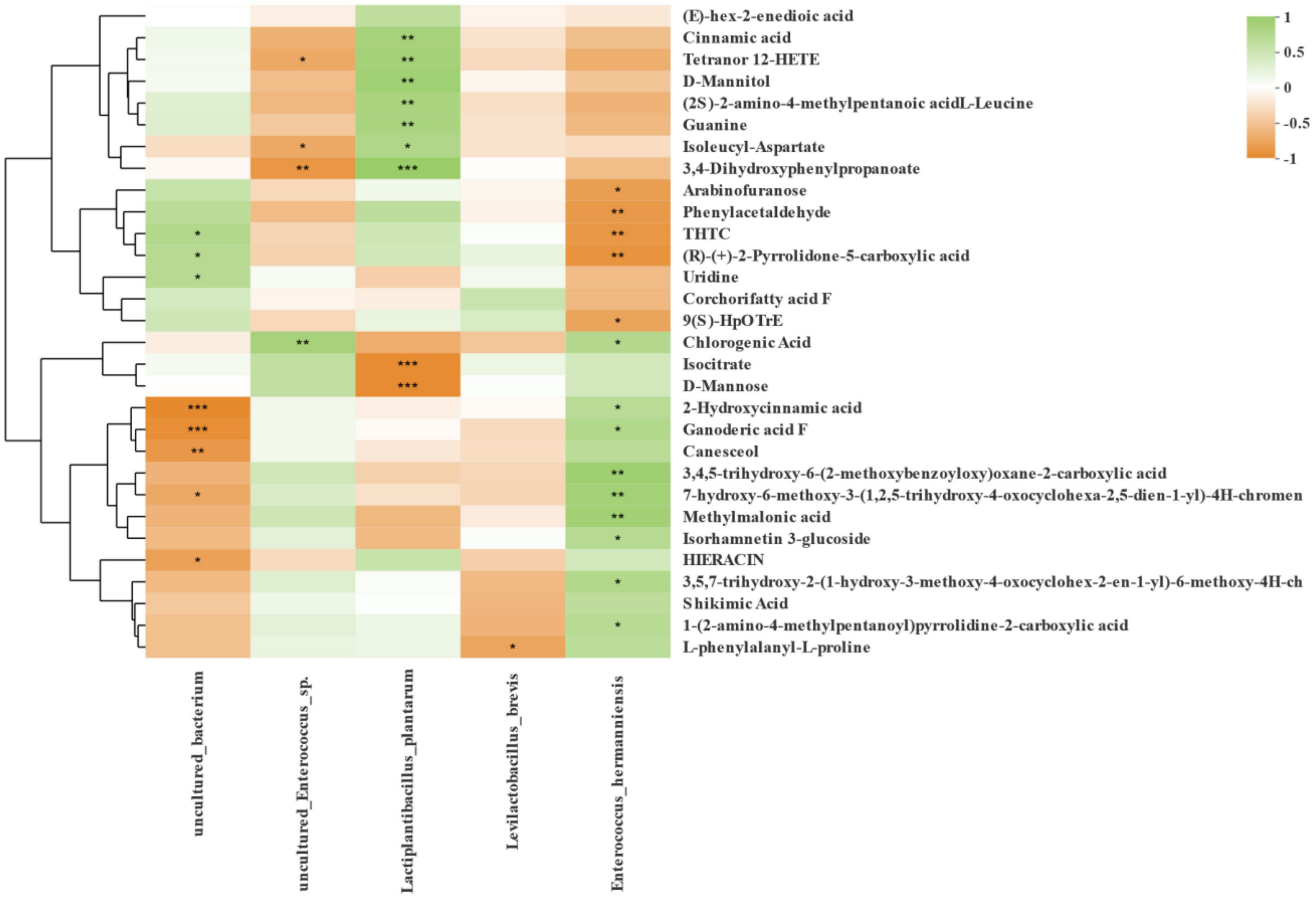
Correlation analysis of high abundance of species-level bacteria and metabolites in silage from three regions. Bacterial community information is displayed horizontally and the fermentation metabolites are displayed vertically. The corresponding value of the middle heat map is the Spearman correlation coefficient r, which ranges between −1 and 1; *r* < 0 indicates a negative correlation (orange), *r* > 0 indicates a positive correction (green), and “*,” “**,” and “***” represent *p* < 0.05, *p* < 0.01, and *p* < 0.001, respectively.

Spearman correlation between bacteria and differential fermentation metabolites at the level of species is shown in Figure 8, and those bacteria had the highly relative abundance in three region silages. *Lactiplantibacillus plantarum* was positively correlated with cinnamic acid, tetranor 12-HETE, D-Mannitol, (2S)-2-amino-4-methylpentanoic acid L-Leucine, guanine, isoleucyl-aspartate and 3,4-Dihydroxyphenyl propanoate, but negatively correlated with isocitrate and D-mannose. *Levilactobacillus brevis* was negatively correlated with L-phenylalanyl-L-proline. The *Enterococcus hermanniensis* was positively correlated with chlorogenic acid, 2-hydroxycinnamic acid, ganoderic acid F, methylmalonic acid, and isorhamnetin 3-glucoside.

## Discussion

Ensiling involves complex interactions between microorganisms and chemical components of raw materials in an anaerobic environment (Wang et al., 2022b). Therefore, the involved species of microorganisms attached to the surface of the raw materials and chemical components of the raw materials are important factors affecting silage quality (Yin et al., 2022). To investigate the contribution of microorganisms and chemical components attached to the raw materials of Italian ryegrass to the fermentation products of Italian ryegrass silage in three different regions, the microbial community characteristics, metabolic pathways and metabolites of Italian ryegrass silages after 60 days ensiling were analyzed.

### Chemical composition and microbial population of raw materials

As a high-quality forage, Italian ryegrass has a good nutritional profile, including high levels of CP and WSC (Yan et al., 2019). In this study, the CP content was higher than 14% DM in all regions, where the CP content of the group SYK was the highest, which was higher than that of perennial ryegrass (10.46% DM, Dong et al., 2020), alfalfa (18.3% DM, Wang et al., 2019), and dried corn stover (6.08% DM, Yan et al., 2019). Guan et al. (2018) reported that differences in temperature, precipitation, and soil environment may influence the CP content. The WSC substance of raw materials is basic for lactic acid fermentation, and the ideal WSC substance ought to surpass 5% DM for a satisfactory silage fermentation (Li et al., 2019). The WSC substance (7.5% DM, 9.01% DM, and 9.23% DM) of fresh Italian ryegrass in the three different regions was higher than 5% DM, thereby demonstrating that adequate substrate may be given for microorganisms. Among these, the CP content (23.54% DM) and WSC content (9.23% DM) in the SYK group were the highest, which may because the SYK region had the highest altitude of the studied regions (Ding et al., 2020; Yang X. et al., 2022). Ding et al. (2020) reported that with an increase in altitude, WSC and CP content also increased in fresh grass. This may because low temperatures at the high altitudes restrain plant respiration, which is conducive to the accumulation of WSC and CP in the cell protoplasm. Wang et al. (2017) suggested that the minimum requirement for LAB number on raw material should be more than 5.00 log_10_ cfu/g FM. In our study, the low number of LAB attached to the raw material may lead to poor silage fermentation.

### Fermentation quality of Italian ryegrass silage in different regions

After 60 days of ensiling, the LAB population of all three groups increased, whereas the WSC contents decreased, suggesting that fermentation of LAB consumes WSC during ensiling. According to Muck et al. (2018), LAB used the WSC as a substrate during fermentation to produced LA and preserve forage during silage. Therefore, the WSC content in each treatment group decreased after ensiling, but all were higher than 5% DM. According to Wang et al. (2022b), higher WSC content in silage may be more beneficial for ruminant nutrition, which also proves that Italian ryegrass silage is a high-quality feed source for ruminants. According to a previous study, a pH of 4.2 or less indicates well-fermented silage (Xiong et al., 2022). In this study, among all treatment groups, the SYK group had the lowest pH at 4.67, which is higher than 4.2. According to previous study (Yan et al., 2019), the pH value of natural fermented Italian ryegrass silage without additives is higher than 4.2, which agrees with our findings. The final pH of silage is affected by several factors (e.g., very high protein and ash content) which may account for silages that present with higher than the normal pH values (Kung et al., 2018). In the SYK group, the CP content of the raw material and the AN/TN ratio after ensiling were higher than others, which indicated that the CP was degraded more during the fermentation process, and the amino acid decarboxylation produced basic amines, resulting in a higher pH value (Pessione et al., 2010). After 60 days of ensiling, the SYK group had the highest LA content, which was possibility due to the relatively high abundance of *Lactiplantibacillus plantarum* (63.98%) and some *Levilactobacillus brevis* (14.28%) in the SYK group. It is reported that homofermentative LAB (e.g., *Lactiplantibacillus plantarum*) produce LA to reduce pH during fermentation, while heterofermentative LAB (e.g., *Levilactobacillus brevis*) produce LA and AA during fermentation to improve aerobic stability (Xu et al., 2021). Silage samples containing heterofermentative LAB had a higher pH and AA concentrations (Xu et al., 2019). However, there was no significant difference in the numbers of LAB in the three different regions, indicating that the fermentation efficiency of LAB in the SYK silage samples may be greater in later fermentation stage (Ding et al., 2020). In our future studies, we can further study the acid-producing ability by screening and purifying the dominant LAB in samples from SYK region. We found that AA and PA content in the SYK group was the highest compared to the other groups, which may be related to the presence of more bacteria producing AA and PA in these raw materials (Guan et al., 2018). Propionibacteria that convert glucose and LA into PA and AA may also exist in SYK silage samples (Kung et al., 2018). The WK group had the highest pH and the lowest LA content, which was due to the lowest relative abundance of *Lactiplantibacillus plantarum* (4.17%) in the WK group (Xu et al., 2021). In addition, compared with the WSC content of the raw materials, the SYK group consumed the most WSC (9.23% DM vs. 5.32% DM) during the fermentation process, and the WK group (7.50% DM vs. 5.25% DM) consumed the least, indicating that the growth and reproduction of *Lactiplantibacillus plantarum* consumed more WSC content (Muck et al., 2018). The ammonia nitrogen ratio is an important indicator of silage fermentation quality; the greater the ratio, the more amino acids and proteins are decomposed (Denek et al., 2016). In the present study, the ammonia nitrogen ratios in all treatments were less than 10%, indicating that the protein degradation of silage was acceptable in all regions (He et al., 2022).

### Microbial community of raw materials and Italian ryegrass silages

Since the ensiling process depends on the interactions of multiple bacteria, the bacterial community structure directly affects the silage quality (Ni et al., 2017). In the present study, the bacteria in all samples were sequenced by SMRT sequencing technology to accurately assess the microbial community and diversity (Table 4). The coverage values were greater than 0.99 in all samples, indicating that the reliability of species detection was very high and the sequencing depth was sufficient to detect the maximum bacterial communities. The alpha diversity indices (ACE, Chao1, and Shannon) were used to reflect the microbial richness and species diversity of samples (Du et al., 2021b). Shannon’s index is used to measure species diversity with a higher value indicating increased species diversity. Ace and Chao 1 indices are used to measure species richness with lower values indicating lower species richness. In our study, the alpha diversity indices (ACE, Chao1, and Shannon) of the fresh material was higher than that of the Italian ryegrass silage samples, and these indices showed trends similar to the Sobs index. This suggests that the microbial richness and species diversity of Italian ryegrass decreased after silage (Cai et al., 2020). This may be because fresh material exists in an aerobic and neutral environment that suits the proliferation of epiphytic aerobic microorganisms (Xu et al., 2021). However, after 60 days of silage, an acidic anaerobic environment was formed in the silage samples, resulting in a decrease in bacterial diversity, which was also confirmed by the lowest pH values and the Shannon index in SYK. This also suggested that the acidic and anaerobic environment affect the succession of microorganisms in the silage (Wang et al., 2022a). Du et al. (2021b) also made the same observations and found that the lowest alpha diversity was observed in high-quality silage due to the large proportion of beneficial microorganisms. Therefore, the alpha diversity index can be used as a reference for evaluating high-quality silage.

Venn diagrams (Figure 2) showed common microbes in different environments for all samples, and the number of unique and shared microbes in different samples based on OTU taxa (Guan et al., 2018). Therefore, the amount of raw material OTUs were different in the three studied regions (Figure 2A). After 60 day of fermentation, the OTUs shared by regions of silage decreased from 99 to 28, and this was due to the formation of an acidic environment by microbial fermentation, which had been the shared OTU of the dominant microbiota, leading to a decrease in the microbial community (Du et al., 2021b).

The PCoA map clearly illustrated the changes of the microbial community before and after ensiling. The microbial community of the raw materials from the three regions was not clearly separated. The distance of SY and A was closer than W, likely because of the similar geographic location and altitude between group AK and SYK, while the geographic location and altitude of the WK group and these two groups were further apart, indicating that the geographic environment, altitude, and climate differences in three different regions affected the microbial composition (Yang X. et al., 2022). The ensiling samples were significantly separated from the raw material samples, indicating that the microbial community was changed during the silage process, which affected the silage fermentation products and metabolic differences (Ni et al., 2017).

The microbial community composition, structure, and function are key to the process of ensiling fermentation (Cai et al., 2020; Du et al., 2022a). In our study, Proteobacteria was the main phyla in Italian ryegrass raw materials in three regions, and this result was same as Wang et al. (2022b). Proteobacteria play an important role in degrading organic matter and accelerating carbon and nitrogen cycles (Ma et al., 2018). After 60 day of fermentation, the relative abundance of Proteobacteria decreased, while Firmicutes increased to become the most dominant phylum. This may be due to the fact that the acidic and anaerobic environment during silage fermentation inhibited the growth and reproduction of Proteobacteria, but promoted the growth and reproduction of Firmicutes (Keshri et al., 2018). According to Wang et al. (2020), the acid hydrolysis function of Firmicutes plays an important role in anaerobic environment, it can produce a variety of enzymes (e.g., proteases).

In the raw material, in addition to unidentified bacteria, we also found several bacteria, such as *Agrobacterium*, *Pantoea*, and *Paracoccus*. According to Gao et al. (2021), imidacloprid biodegradation in soils was aided by *Paracoccus* bacteria and it was positively correlated with high degradation activity. According to Walterson and Stavrinides (2015), *Pantoea* was isolated from *Enterobacter* genus and fermented lactose while forming slime colonies, and some *Pantoea* sp. are pathogenic to vegetables (Du et al., 2022a). However, little research has been done on the role of these bacteria in silage. Ogunade et al. (2018) reported *Pantoea* had an ability to reduce ammonia-nitrogen concentrations in silage, but more studies are required to elucidate their role during ensiling. Based on previous study (Gao et al., 2021), we speculate that these bacteria may originate from the soil in which the Italian ryegrass raw material lives and are carried from the soil during the growth or harvest, but the role of these bacteria in silage fermentation needs further study.

After 60 day of ensiling, *Lactiplantibacillus* and *Enterococcus* were the most dominant genera, but their relative abundances varied among treatments. Previous study (Wang et al., 2019) have found that *Lactiplantibacillus* was the most common bacteria after successful fermentation of silage, because *Lactiplantibacillus* inhibits the growth of harmful microorganisms by producing LA and lowering the pH of the silage under anaerobic conditions (Cai et al., 2020). In the current study, the SYK group had the highest relative abundance of *Lactiplantibacillus plantarum*, followed by the AK group, and WK had the lowest relative abundance of *Lactiplantibacillus plantarum*. The SYK group had the lowest abundance of *uncultured Enterococcus* sp., and the WK group had the highest relative abundance of *uncultured Enterococcus* sp. and uncultured bacterium, which indicated that during the fermentation process of SYK, the attached microorganisms that were conducive to fermentation interacted with the chemical components in the raw materials to promote fermentation, thereby increasing the abundance of LAB. However, LAB in the WK group were not fully fermented, resulting in a lower abundance of *Lactiplantibacillus plantarum* and a higher abundance of uncultured *Enterococcus* sp. and uncultured bacterium (Du et al., 2021c). Moreover, the WK group had the highest pH value, followed by the AK group, and the SYK group had the lowest pH value. This was because *Lactiplantibacillus plantarum* is a facultatively homofermentative LAB that grows well in acidic environments and promotes LA fermentation during silage (Li and Nishino, 2013). Santos et al. (2016) reported that most *Enterococcus* sp. detected in silage were non-pathogenic bacteria that competed with LAB for nutrients to utilize the WSC as a substrate to survive at low pH environment, resulting in nutrient loss.

### Correlation analysis of fermentation products and microbial community

Microorganisms influence silage fermentation through a series of metabolites, and these metabolites also affect the community structure of microorganisms. They interact during the fermentation process to jointly regulate the process of silage fermentation and affect silage quality (Du et al., 2022b). In the current study, we performed correlation analyses between the bacterial community and terminal fermentation products at the species level (Figure 5) by using SMRT. After 60 day of ensiling, *Lactiplantibacillus plantarum*, *Latilactobacillus sakei*, and *Weissella paramesenteroides* were negatively correlated with pH, but positively correlated with AN/TN, LA, AA, and PA. *Enterococcus faecalis* and uncultured *Enterococcus* sp. was positively correlated with pH, but negatively correlated with LA, AA, and PA. The results are similar to those of Du et al. (2022b), indicating that acid tolerance of *Lactiplantibacillus plantarum*, *Latilactobacillus sakei*, and *Weissella paramesenteroides*, and their critical importance for LA production during fermentation. *Lactiplantibacillus plantarum* rapidly acidifies silage to inhibit the growth of harmful microorganisms in the late stage of ensiling (Du et al., 2021b). *Latilactobacillus sakei* was able to use glucose, fructose and different hexose sugars as primary energy sources to produce LA through the glycolytic pathway (Belleggia et al., 2022). In addition, *Latilactobacillus sakei* was degraded by proteases and aminopeptidases, using free amino acids and nucleotides as energy sources (Belleggia et al., 2020). Du et al. (2021b) reported that *Lactiplantibacillus plantarum* can reduce the pH of the silage and limit the loss of protein and carbohydrates, thereby reducing the NH_3_-N concentration and the fermentation loss in the silages. Generally, LAB fermentation can effectively inhibit protein decomposition, whereas the presence of LAB had the opposite effect in this study. This may be because the initial acidification caused by lactic acid fermentation fails to effectively prevent the proliferation and fermentation of Clostridia in silage, resulting in poor quality silage due to the production of propionic acid and butyric acid or the accumulation of ammonia and amine (Li et al., 2020). Tsigkrimani et al. (2022) reported that *Enterococcus faecalis*, *Weissella paramesenteroides*, and *Enterococcus* sp. were resistant to antibiotics (vancomycin). These microorganisms affect animal health by affecting silage quality, which would be an interesting research topic.

### KEGG metabolomic pathways and properties of Italian ryegrass silage

Metabolomics technology can accurately identify metabolites in silage. In recent years, many studies (Guo et al., 2018; Xu et al., 2019, 2021) have applied metabolomics technology to identify metabolites during silage fermentation and the correlation between fermenting bacteria and metabolites. At the same time, the KEGG database was used to analyze the biological function and utility of metabolites from a high-level and genomic perspective (Wang et al., 2022a). This can be achieved by displaying the metabolites in the metabolic concentration on the KEGG pathway map, and using KEGG enrichment analysis to explore the most relevant pathways and underlying mechanisms (Yang Z. et al., 2022).

The ensiling process is mediated by microbial metabolic pathways, which have transformed metabolites or degraded substrates (Bai et al., 2022). In this study, we constructed a metabolic set of the identified differential metabolites, and used KEGG enrichment analysis to clarify that interaction between the microorganisms on the surface of Italian ryegrass and the chemical components of Italian ryegrass in three regions had extremely significant effects on biosynthesis of other secondary metabolites (isoquinoline alkaloid biosynthesis, betalain biosynthesis), amino acid metabolism (alanine, aspartate and glutamate metabolism, tyrosine metabolism, tryptophan metabolism), carbohydrate metabolism (glyoxylate and dicarboxylate metabolism, citrate cycle, amino sugar and nucleotide sugar metabolism), membrane transport (ABC transporters), nucleotide metabolism (pyrimidine metabolism) and energy metabolism (nitrogen metabolism). In addition, the biosynthesis of other secondary metabolites (isoquinoline alkaloid biosynthesis), amino acid metabolism (tyrosine metabolism) and carbohydrate metabolism (citrate cycle) were the most significant and important metabolic pathways in the SYK, AK, and WK groups, respectively.

Du et al. (2022a) reported that glycolysis, protein hydrolysis, carbohydrate metabolism, and amino acid metabolism are the main microbial metabolic pathways affecting the flavor and quality of silage. Amino acids are essential substances for forage grasses and its metabolism can be oxidized to carbon dioxide and water through the tricarboxylic acid cycle, releasing energy, and playing an important role in protein synthesis and primary metabolism of forage grasses (Du et al., 2021b, 2022b). Amino acid metabolism plays a crucial role in the formation of metabolites, most of which are essential metabolites required by LAB for growth and protein synthesis during fermentation (Zhou et al., 2022). Phenylalanine is an essential and aromatic amino acid that is oxidized to tyrosine in the body phenylalanine hydroxylase and is involved in sugar metabolism (Pan et al., 2020). In the citric acid cycle, a condensation reaction between oxaloacetic acid and the acetyl group of acetyl-CoA produces citric acid, which is often used as a flavoring agent in foods, and Citric acid-mediated pH adjustment can improve oxidant performance and enzyme activity, thus extending food shelf life (Ke et al., 2017). Understanding the metabolic pathways and biological functions of metabolites can help us regulate the fermentation process to obtain high-quality silage or silage with certain biological functions.

### Correlation analysis of high abundance bacteria and fermentation metabolites

Previous research (Guo et al., 2018; Xu et al., 2019; Guan et al., 2020) found that metabolomics technology can more accurately mirror the metabolites composition of the environment, and that it can also be used in silage. Those studies also highlighted links between amino acid metabolism, coenzyme factors and vitamin metabolism, lipid metabolism, carbohydrate metabolism, and terpenoid and ketone compound metabolism in Napier grass silage inoculated by screened LAB. The above studies have greatly enriched our understanding of metabolites in silage. In this study, we also used SMRT sequencing and metabolomics technology to analyze the correlation of differential metabolites in each treatment group with bacteria with higher relative abundance. The contents of cinnamic acid, tetranor 12-HETE, D-Mannitol, (2S)-2-amino-4-methylpentanoic acid L-Leucine, guanine, isoleucyl-aspartate and 3,4-Dihydroxyphenyl propanoate were positively correlated with the abundance of *Lactiplantibacillus plantarum*, while the contents of isocitrate and D-mannose were negatively correlated with the abundance of *Lactiplantibacillus plantarum*. Xu et al. (2021) reported that *Lactiplantibacillus plantarum* was positively correlated with 3-hydroxy fatty acids and could be considered as a species for screening inoculants with potential antifungal activity. Cinnamic acid and its derivatives are widely found in plants, grains, and vegetables, and in recent years, they have attracted much attention due to the positive health effects of various derivatives of cinnamic acid (such as caffeic acid, ferulic acid), including antioxidant, antibacterial and hepatoprotective effects (Yamaga et al., 2022). Ferulic acid, also a hydroxycinnamic acid, has antioxidant activity and is negatively correlated with *Lactiplantibacillus plantarum* (Xu et al., 2021). The content of L-phenylalanyl-L-proline was negatively correlated with the abundance of *Levilactobacillus brevis*. L-phenylalanyl-L-proline is a derivative of L-proline, a major amino acid that maintains cell structure and function, as well as a key regulator of cell metabolism and physiology (Liu et al., 2019). The contents of chlorogenic acid, 2-hydroxycinnamic acid, ganoderic acid F, methylmalonic acid and isorhamnetin 3-glucoside were positively correlated with the abundance of *Enterococcus hermanniensis*. Chlorogenic acid is a phenylpropionic acid compound with antioxidant, anticancer, antibacterial, antihistamine and other health promoting properties (Zhou et al., 2022). Given that *Enterococcus hermanniensis* was positively correlated with chlorogenic acid, we may consider using it as a candidate strain to produce LAB with antibacterial properties in future study. An in-depth understanding of the relationship between metabolites and fermenting bacteria will help us to understand the fermentation mechanism of silage from the perspective of bioinformatics, so as to provide a theoretical basis for screening functional lactic acid bacteria and producing functional compound lactic acid bacteria silage additives.

## Conclusion

The different regional environments can change the microbial community attached to the surface of Italian ryegrass and the chemical composition of Italian ryegrass, thereby altering the fermentation quality and metabolomic characteristics of silage by affecting the interaction between microorganisms and chemical components. After silage, the SYK samples had the lowest pH value and best fermentation quality. The biosynthesis of other secondary metabolites (isoquinoline alkaloid biosynthesis), amino acid metabolism (tyrosine metabolism) and carbohydrate metabolism (citrate cycle) were the most significant and important metabolic pathways in the SYK, AK, and WK groups, respectively. Cinnamic acid content, which has a positive effect on health, increased with the abundance of *Lactiplantibacillus plantarum*, and chlorogenic acid and 2-hydroxycinnamic acid, which have antibacterial and antioxidant properties, increased with the abundance of *Enterococcus hermanniensis*. Therefore, using SMRT and metabolomics technology to conduct multi-omics combined analysis of silage will help us to deeply understand the fermentation mechanism of silage. Furthermore, our findings provide new ideas and information for the screening of functional lactic acid bacteria strains and the production of functional compound additives and high-quality functional silage.

## Data availability statement

The datasets generated for this study can be found in the NCBI and an accession number SRP373166.

## Author contributions

ZF: methodology, visualization, validation, data curation, and wrote the original draft. LS and CG: interpreted the data and edit the language. ZW: conceptualization, acquisition, and writing – review and editing. YL and JH: software. GG: conceptualization and funding acquisition. All authors have read and agreed to the published version of the manuscript.

## Funding

This work was supported by the Key Laboratory of Forage Cultivation and the Processing and Highly Efficient Utilization of the Ministry of Agriculture, the Key Laboratory of Grassland Resources of the Ministry of Education, and funded by the National Technical System of Forage Industry for Dry Grass Storage (CARS-34), China.

## Conflict of interest

The authors declare that the research was conducted in the absence of any commercial or financial relationships that could be construed as a potential conflict of interest.

## Publisher’s note

All claims expressed in this article are solely those of the authors and do not necessarily represent those of their affiliated organizations, or those of the publisher, the editors and the reviewers. Any product that may be evaluated in this article, or claim that may be made by its manufacturer, is not guaranteed or endorsed by the publisher.

## Footnotes

^1^ https://cloud.majorbio.com

^2^ http://www.genome.jp/kegg/

^3^ https://docs.scipy.org/doc/scipy/

^4^ www.i-sanger.com

## Abbreviations

LAB: lactic acid bacteria
WSC: water-soluble carbohydrates
LA: lactic acid
SMRT: Single Molecule Real Time
UHPLC–MS/MS: ultra-high performance liquid chromatography-mass spectrometry
FW: fresh matter
DM: dry matter
TN: total nitrogen
AN: ammonia nitrogen
CP: crude protein
NDF: neutral detergent fiber
ADF: acid detergent fiber
AA: acetic acid
PA: propionic acid
BA: butyric acid
OTUs: operational taxonomic units
PCoA: principal coordinate analysis
PCA: principal component analysis
PLSDA: partial least-square discriminant analysis
OPLS-DA: orthogonal projections to latent structure discriminant analysis
KEGG: Kyoto Encyclopedia of Genes and Genomes
WK: Italian ryegrass silage from Ordos (China, Inner Mongolia, Ordos-wushenqi)
AK: Italian ryegrass silage from Hohhot (China, Inner Mongolia, Hohhot)
SYK: Italian ryegrass silage from Ulanqab (China, Inner Mongolia, Ulanqab-siziwangqi)
